# A Multicentric Brazilian Investigative Study of Copy Number Variations in Patients with Congenital Anomalies and Intellectual Disability

**DOI:** 10.1038/s41598-018-31754-2

**Published:** 2018-09-06

**Authors:** J. R. M. Ceroni, R. L. Dutra, R. S. Honjo, J. C. Llerena, A. X. Acosta, P. F. V. Medeiros, M. F. Galera, É. A. Zanardo, F. B. Piazzon, A. T. Dias, G. M. Novo-Filho, M. M. Montenegro, F. A. R. Madia, D. R. Bertola, J. B. de Melo, L. D. Kulikowski, C. A. Kim

**Affiliations:** 1 Unidade de Genética, Departamento de Pediatria, Instituto da Criança, Hospital das Clínicas da Faculdade de Medicina da USP, HCFMUSP, São Paulo, SP Brazil; 2 Laboratorio de Citogenômica, Departamento de Patologia, Faculdade de Medicina da USP, FMUSP, São Paulo, SP Brazil; 3 Instituto Nacional de Saúde da Mulher, da Criança e do Adolescente Fernandes Figueira - Fiocruz, Rio de Janeiro, RJ Brazil; 4 Universidade Federal da Bahia, Salvador, BA Brazil; 5 Universidade Federal de Campina Grande, Campina Grande, PB Brazil; 6 Universidade Federal do Mato Grosso, Cuiabá, MT Brazil; 7 Centro de Pesquisa sobre o Genoma Humano e Células-Tronco, Instituto de Biociências, Universidade de São Paulo, São Paulo, SP, Brazil., São Paulo, SP Brazil; 8 Laboratório de Citogenética e Genómica - Faculdade de Medicina, Universidade de Coimbra, CIMAGO - Centro de Investigação em Meio Ambiente, Genética e Oncobiologia, Faculdade de Medicina, Universidade de Coimbra, Faculdade de Medicina, Universidade de Coimbra, CNC, IBILI - Faculdade de Medicina, Universidade de Coimbra, Coimbra, Portugal

## Abstract

Genomic imbalances are the most common cause of congenital anomalies (CA) and intellectual disability (ID). The aims of this study were to identify copy number variations (CNVs) in 416 patients with CA and ID from 5 different genetics centers within 4 different states by using the Multiplex Ligation-dependent Probe Amplification (MLPA) technique and to apply the chromosomal microarray (CMA) methodology in selected cases. The samples were analyzed by MLPA kits P064, P036, P070 and P250. Positive results were found in 97/416 (23.3%) patients. CMA was applied in 14 selected cases. In 6/14 (42.85%) patients, CMA detected other copy number variations not detected by the MLPA studies. Although CMA is indispensable for genotype refinement, the technique is still unfeasible in some countries as a routine analysis due to economic and technical limitations. In these cases, clinical evaluation followed by karyotyping and MLPA analysis is a helpful and affordable solution for diagnostic purposes.

## Introduction

Genomic imbalances are the most common cause of congenital anomalies (CA) and intellectual disability (ID). The first association was demonstrated almost 60 years ago by peripheral blood karyotyping in a patient with trisomy 21^1^. Currently, the vast majority of CA and ID cases remain without diagnosis after karyotyping due to technical limitations, particularly due to the inability to detect genomic variations smaller than 3–5 Mb and the subjectivity of the interpretation by cytogeneticists. For this reason, G-band karyotyping is indicated in the investigation of only selected cases – clinical suspicion of aneuploidies, balanced translocations, and low-level mosaicism (<10%).

CNVs are defined as DNA fragments larger than one kilobase (1 Kb), with a distinct number of copies in the genome^2^. Depending on the clinical relevance of the imbalance, CNVs are classified as pathogenic, benign, or of uncertain clinical significance (VOUS)^3,4^. The corresponding phenotypes of CNVs depends on the region involved in the rearrangement, the genes affected, dosage effects, gene rupture, recurrence in healthy individuals, known pathogenicity in the literature, pattern of inheritance, and the influence of the imbalance over the expression of the other allele^5,6^, as there is higher susceptibility of other genomic rearrangements in these patients^7^.

Until the advent of molecular techniques such as fluorescent *in situ* hybridization (FISH), polymerase chain reaction (PCR), Multiplex Ligation Probe-dependent Amplification (MLPA), and chromosomal microarray (CMA), small CNVs were not detected^8^. Consistent with this fact, the literature has shown a higher diagnostic usage of cytogenomic techniques over G-band karyotyping in patients with CA and ID^9^, with a 9–30% detection by MLPA, depending on the combination of kits and previous screening^10,11^, and 20% by chromosomal microarray (CMAs)^12^ versus 5% with G-band karyotyping.

Despite the fact that some pathogenic CNVs cause syndromes with a clinically recognizable phenotype, such as the 7q11.23 microdeletion (Williams syndrome) and the 22q11.23 microdeletion (DiGeorge syndrome), that could be investigated by a specific genetic test (e.g., FISH), the vast majority of small CNVs produce a nonspecific phenotype, for which CMA is the recommended genetic test. Since 2008, the substitution of karyotyping for CMA has been recommended by both the First International Workshop on Uniform Cytogenetic Arrays and Shared Databases and the Canadian Society for Medical Laboratory Science. Therefore, CMA is the gold standard technique for the detection of small CNVs, defined as fragments larger than one kilobase (1 Kb), with a distinct number of copies in the genome^13^, and not detectable by karyotyping (<5 Mb). CNVs in the human genome are the main cause of CA (2–3% of live births) and ID^13,14^.

While very valuable in routine diagnosis, CMA is not available in the public healthcare systems of underdeveloped countries due to a lack of economic and human resources. Therefore, the establishment of less expensive alternatives based on the patients’ clinical presentation and cost-benefit methodology are of great interest in clinical practice. This is particularly true for congenital heart defects, which are the most prevalent category of birth defects^15^. The application of MLPA studies in this group of patients provides a high diagnostic yield^16^, suggesting a good cost-benefit.

Thus, in order to determine the presence of CNVs in Brazilian patients with CA, ID and/or neurodevelopmental delay whose peripheral blood G-banding karyotyping had normal results, we used a combination of MLPA kits, and CMA was applied only in selected cases. We also evaluated the efficiency and applicability of this cytogenomic approach in the investigation of a large series of patients undergoing clinical genetic follow-up.

## Results

Molecular analysis by MLPA detected pathogenic CNVs in 97/416 patients (23.3%). In 59 (14.1%) patients, kit P064 was positive; in 45 (10.8%) patients, kit P036 was positive; in 43 (10.3%) patients, kit P070 was positive; and in 4 (0.9%) patients, kit P250 was positive. Table 1 is illustrative of these results and shows additional information of the patients.

**Table 1:** Genomic imbalances found by MLPA within the use of different kits.

MLPA Kit	Positive Results	Mean Age	Sex
P036	47/416 (11.3%)	N/A	N/A
P064	59/416 (14.2%)	N/A	N/A
P070	47/416 (11.3%)	N/A	N/A
P250	4/416 (0.9%)	N/A	N/A
P036 + P070	34/416 (8.2%)	N/A	N/A
P036 + P070 + P064	13/416 (3.1%)	N/A	N/A
TOTAL	97/416 (23.3%)	10.5 years	172 (41.3%) males/244 (58.7%) females

**Figure 1:**
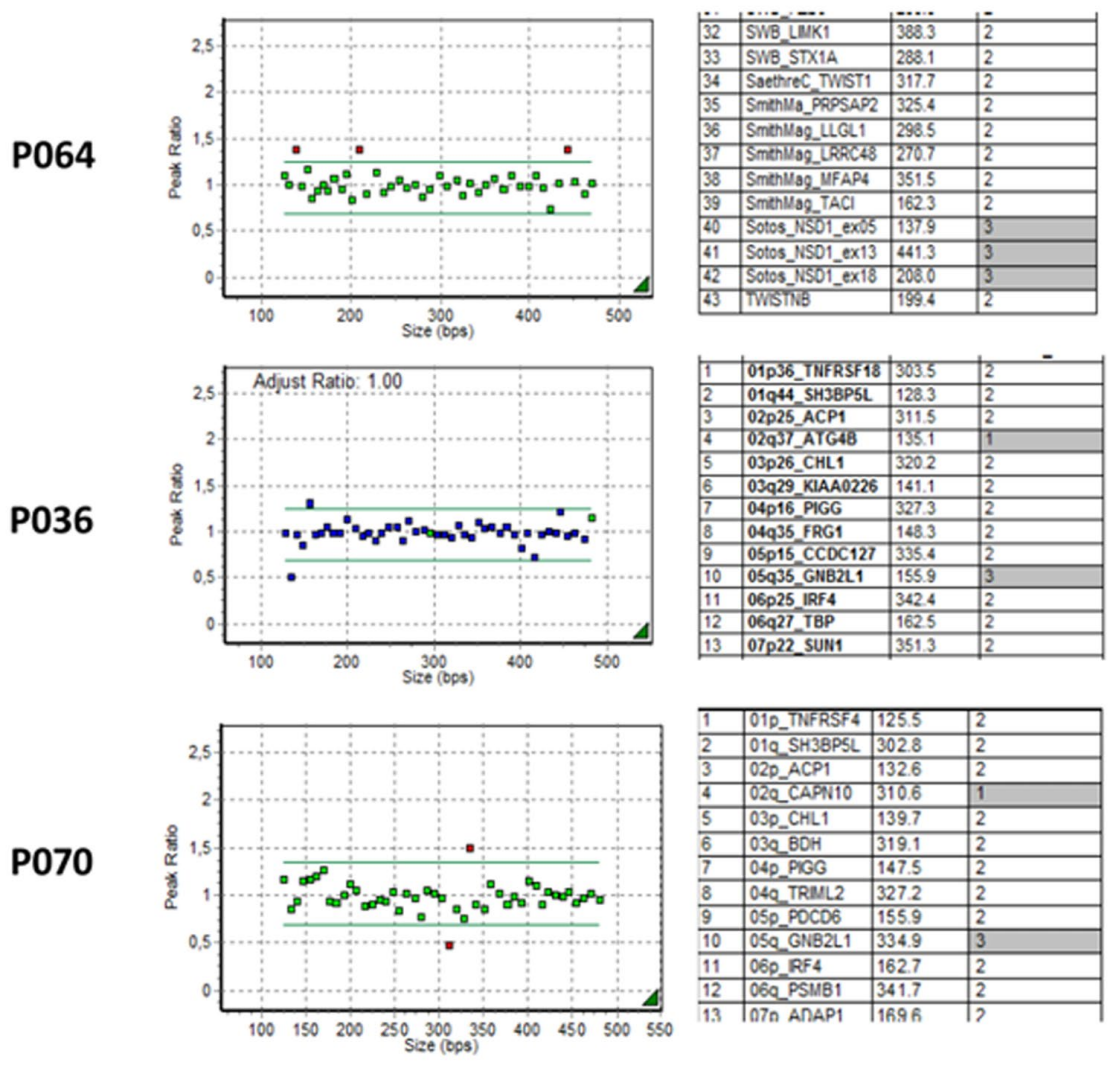
MLPA showing 5q35.3 microduplication and 2q37.3 microdeletion.

Recurrent chromosomal imbalances were identified as microdeletion 22q11.23 (DiGeorge syndrome) in 26 patients, including 23 with the typical 3 Mb deletion and 3 with atypical deletions. Microdeletion 7q11.23 (Williams-Beuren syndrome) was identified in 20 patients, microdeletion 1p36 was identified in 6 patients, and microdeletion 4p16.3 (Wolf-Hirschhorn syndrome) was identified in 5 patients. Other imbalances found are shown in Table 2. In 24/97 (24,7%) patients, the MLPA detected more than 1 CNV (Table 2). Figure 1 demonstrates the results of a patient with two concurrent CNVs.

**Table 2:** Other chromosomal imbalances detected by MLPA technique.

Chromosomal imbalance detected by MLPA	Number of patients
microduplication 2p25.3/microdeletion 4q35.2	1
microdeletion 2q37.3/microduplication 5q35.3	5
microduplication 3q29/microdeletion 9p24.3	1
microdeletion 4p16.3/microduplication 8p23.3	1
microdeletion 4q35	2
microdeletion 4q35.2/microduplication 5q35.3	1
microduplication 4q35.2/microdeletion 7q36.3	1
microduplication 4q35.2/microduplication Xq28	1
microdeletion 5p15.3	3
microduplication 5p15.3/microduplication 14q32.3	1
microduplication 5p15.3/microdeletion Xq28	1
microduplication 5q	3
microdeletion 7p22.3/microduplication 12q24.33	1
microduplication 7q11.23	3
microdeletion 7q11.23/microduplication Xp22/microduplication Xq28	1
microdeletion 9p24.3/microduplication 18q23	2
microduplication 9p24.3	2
microduplication 11p15.5	1
microduplication 12p13.33	1
microduplication 12q24.3/microduplication 15q11.2	1
microduplication 15q26.3/microdeletion Xq28	1
microdeletion 15q11	1
microduplication 15q11	1
microduplication 16q24.3	1
microdeletion 17p11	2
microdeletion 17p13.3/microduplication 17q25.3	1
microdeletion 20q13	1
microduplication 22q11.1	3
microduplication 22q13.3	1
microdeletion 17p13.3/microduplication 17q25.3	1
microdeletion 20q13	3
microduplication 22q11.21/microdeletion 22q13.33	1
microdeletion Xp22.33/microdeletion Xq28	1
microduplication Xp22.33	1
microduplication Yp11.32/microduplication Yq28	1

CMA studies were performed in selected 14 patients in order to better delineate genotype. These patients were selected by two main criteria, in addition to the availability of financial resources: presence of more than one CNV detected by MLPA and/or atypical CNVs. In 6/14 (42.8%) patients, CMA identified additional CNVs not detected by MLPA, which are described as below and shown in Table 3.

**Table 3:** Comparision between MLPA vs. array results performed on the same patient.

No. of patients	MLPA	Array
2	KIT P036, P064 – 2q37 (x1) KIT P036, P064, P070 – 5q35.3 NSD1 (x3)	Oligoarray – arr[hg19] 5q35.1-35.3 (172,194,873–180,705,539)x3 ~8.5 Mb Oligoarray – arr[hg19] 2q37.3 (239,550,182–243,029,573)x1 ~3.5 Mb
1	KIT P036, P064 – 2q37 (x1) KIT P036, P064, P070 – 5q35.3 NSD1 (x3)	Oligoarray – arr[hg19] 5q35.1-35.3 (172,194,873–180,705,539)x3 ~8.5 Mb Oligoarray – arr[hg19] 2q37.3 (239,550,182–243,029,573)x1 ~3.5 Mb Oligoarray – arr[hg19] 12p13.31 (7,986,563–8,123,306)x1 ~136,7 Kb (BENIGN) Oligoarray – arr[hg19] 18q12.1 (27,815,609-28,057,171)x3 ~241 Kb (BENIGN)
1	KIT P036, P064, P070 - 5q34 NSD1 (x3) KIT P036, P064, P070 - 4q34 TRIML2 (x1)	CGH-array – arr[hg19] 5q34-35.3 (160,148,716–180,712,253)x3 ~20 Mb CGH-array – arr[hg19] 4q34.3–35.2 (179,962,284–190,790,881)x1 ~10.8 Mb
1	KITS P036, 070 – 5p15 PDCD6, CCDC127 (x3) KITS P036, 070 – 14q11.2 CCNB1IBP1 (x3)	CGH-array - arr[hg19] 5p15.33-p13.1 (37,692–33,434,546)x3 ~33.4 Mb CGH-array - arr[hg19] 14q11.2-q12 (19,361,358–25,127,451)x3 ~5.8 Mb
3	KIT P036 - 2q37 ATG4B (x1) 5q36 GNB2L1 (x3) KIT P070 - 2q CAPN10 (x1) 5q GNB2L1 (x3) KIT P064 - 5q35.3 NSD1 (x3)	SNP-array – arr[hg19] 2q37.3(239,550,182–243,029,573)x1 ~3.47 Mb SNP-array – arr[hg19] 5q35.1-q35.3(172,194,873–180,705,539)x3 ~8.5 Mb SNP-array – arr[hg19] 18q12.1(27,815,609–28,057,171)x3 ~241 Kb (BENIGN)
2	KITS P036, P070 - 9p24.3 DOCK8 (x1) KITS P036, P070 - 18q23 CTDP1 (x3)	CGH-array - arr[hg19] 9p24.3-p24.2 (199,953–4,336,197)x1 ~4.17 Mb CGH-array - arr[hg19] 18q12.3-q23 (39,129,720–78,012,829)x3 ~38.88 Mb
1	KITS P064, P029 - 7q11.23 FZD9 (x3)	Oligoarray - arr[hg 19] 18p11.21 (14,550,022–14,823,578)x1 ~273.6 Kb (BENIGN)
1	KIT P036, P070 – Xp22 SHOX (x3), Xq28 SYBL1 (x3) KIT P064 – 7q11.23 CYLN2a, CYLN2b, STX1a, ELN, LIMK1 (x1)	SNP-array – arr[hg19] Chr7(72,569,012–72,685,658)x1 ~116 Kb SNP-array – arr[hg19] Chr7(73,052,174–74,628,459)x1 ~1.57 Mb SNP-array – arr[hg19] Chr7(33,135,610–33,193,680)x3 ~58 Kb SNP-array – arr[hg19] Chr13(94,579,078–94,639,273)x1 ~60 Kb (BENIGN) SNP-array – arr[hg19] ChrX(154,997,451–155,240,482)x3 ~243 Kb SNP-array – arr[hg19] ChrX(198,061–2,693,037)x3 ~2.5 Mb
1	KIT P250 - 22q11.2 SNAP29, LZTR1 (x1) ~340 Kb KIT P064 - 22q11.2 SNAP29 (x1)	SNP-array - arr[hg19] 22q11.2 (21,069,073–21,608,479)x1 ~539.4 Kb
1	KIT P036, P070 - 1p36 TNFRSF4, TNFRSF18 (x1)	Oligoarray - arr[hg19] 1p36.3(564,620–2,456,203)x1; ~1.89 Mb Oligoarray - arr[hg19] 1p36.3(2,473,257–3,446,813)x1; ~973 Kb Oligoarray - arr[hg19] 1p36.3(3,474,630–3,641,681)x3 ~167 Kb

In three patients, array confirmed the CNVs found in MLPA (microdeletion 2q37 and microduplication 5q35.3) and demonstrated a benign duplication of 241 Kb in chromosome 18q12 that could not be detected by the combination of MLPA kits used because there were no probes at the specific regions.

**Figure 2:**
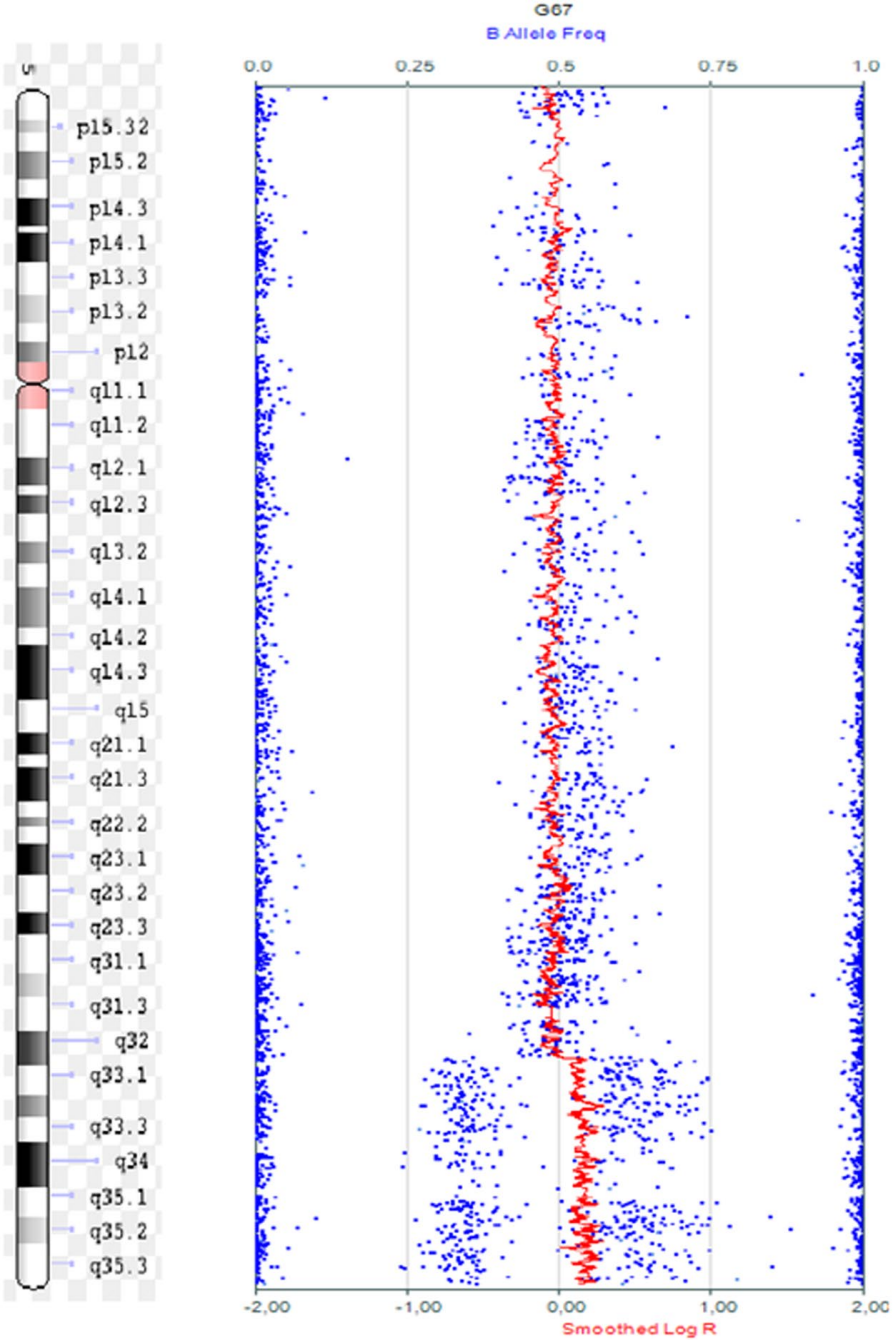
SNP-array showing duplication 5q34q35.3 (160,148,716–180,712,253)x3.

In one patient, array confirmed the CNVs found by MLPA (microdeletion 2q37 and microduplication 5q35.3 – shown in Fig. 2) and detected an additional two CNVs: one deletion of 136.7 Kb in chromosome 12q13 and a duplication of 241 Kb in chromosome 18q12, both classified as benign. Neither of these deletions could be detected by the combination of MLPA kits utilized.

One patient had MLPA results demonstrating two microduplications in chromosome X (Xp22 and Xq28), and a microdeletion of chromosome 7q11.23; however, array results of this patient showed a complex rearrangement in chromosome 7q11.23, two duplications in chromosome X (243 Kb in Xq28 and 2.5 Mb in Xp22.33) and an additional CNV not detected by MLPA, a 60-Kb microdeletion on chromosome 13q31.3.

One patient had MLPA results that showed a microdeletion in chromosome 1p36, and CMA results showed a complex rearrangement in chromosome 1p36.3, two microdeletions of 1.89 Mb and 973 Kb, and a microduplication of 167 Kb.

Parental studies revealed CNVs were *de novo* in 59/97 patients (60.8%), inherited in 8 (8.2%) patients and classified as of unknown origin in 30 (31%) patients due to the absence of a parental DNA sample.

## Discussion

In patients with isolated ID, subtelomeric CNVs can be present in up to 3–6% of cases, and when ID is associated with any CA, CNVs are found in up to 10%^17,18^ of cases. In patients with congenital heart diseases, recent work has shown that CNVs are present in 25.5% of patients^16^. Genetic syndromes that present with congenital heart defects were prevalent in our cohort, including microdeletion 22q11.2 in 26 patients (22 with the typical deletion) and Williams-Beuren syndrome in 20 patients. Both of these syndromes are characterized by congenital heart defects - conotruncal malformation and supravalvar aortic stenosis, respectively.

Subtelomeric regions are more likely to be involved in rearrangements because only one chromosomal breakpoint is required^19^. Subtelomeric chromosomal imbalances found by the combination of MLPA kits P036 and P070 were found in 47 (11.3%) patients, similar to the numbers reported in the literature^20–22^. Vries *et al*.^23^ demonstrated that 4.8% of 2500 patients with ID had CNVs in subtelomeric regions. Ravnan *et al*.^24^ studied subtelomeric regions of 11,688 blinded cases and observed pathogenic variants in 2.6% of patients.

A total of 14.1% of patients (59/416 patients) tested positive with P064, higher than that observed in the literature. Kirchhoff *et al*. 2007 showed that 6% of 258 patients with ID were positive when evaluated by MLPA kit P064. This finding reinforces is consistent with the fact that higher diagnostic sensitivities are obtained when the cohort consists of patients with congenital heart defects that have had previous assessment by clinical geneticists.

The combination of kits P064, P036 and P070 in our study was positive in 97/416 (23.3%) patients. In our previous study of 261 patients by Jehee *et al*.^10^, pathogenic imbalances were found in 21.8% patients. Both results show higher sensitivity when compared to other studies^11,25^.

In our study of 416 patients from five different genetic centers, MLPA confirmed the diagnosis in 97(23,3%) patients and was complemented with CMAs in 14 selected patients (Table 3). Indeed, despite the huge evolution of genomic testing in recent years, a detailed clinical evaluation retains tremendous significance because it allows for the proper selection of patients and genetic test, thereby raising the cost-benefit ratio. In underdeveloped countries, this is essential to the sustainability of the healthcare system.

CMA leads to a better characterization of genomic imbalances, genotype-phenotype correlation, and the identification of candidate genes to relevant phenotypes^26^. Oligoarrays have been used in genomic evaluation of patients with ID and CA. Because most oligoarrays detect submicroscopic CNVs throughout the whole genome^27,28^ with high resolution (~0.7 Kb depending on the platform and probe distribution within the genome)^29–31^, these investigations have shown that submicroscopic chromosomal imbalances affect transcription dosage, sequence, structure and function of different genes^7,32^. Arrays also have the capability of detecting complex rearrangements.

Although precise breakpoints were only detectable with CMA, eight (57.14%) patients did not show other CNVs. Of the six (42.86%) patients with discrepant results, 4 had CNVs that could not be detected by the MLPA kits used but were all classified as benign. Therefore, despite the fact that without CMA these CNVs would have gone unnoticed, none had clinical significance. The two cases in which clinically relevant CNVs were detected by CMA and not by MLPA were due to complex chromosomal rearrangements that are not expected to be detectable by MLPA.

The array of one patient, whose clinical features resembled Williams-Beuren syndrome but with atypical behavior and karyotype 46, XYY [20], showed complex rearrangements in chromosomes 7 and 13 and a small deletion in the X chromosome^33,34^.

One patient had conflicting results, in which MLPA detected duplication in chromosome 7q11.23, but CMA demonstrated only a benign microdeletion of 273.6 Kb in chromosome 18p11.21. One possibility for this discrepancy is sample exchange. Further studies have not been performed in this patient.

In our patients, CNVs were inherited in 8 cases, and none of the carriers presented relevant symptoms. Incomplete penetrance of CNVs, broad phenotype expressivity^10,35^, imprinting effects, and a second mutation not detectable by microarray on the other allele are plausible explanations for lack of clinical findings.

In summary, considering the relative low cost of the technique and the detection of alterations in 23.3% of patients, the investigation of CNVs in our patients with CA and ID by multiplex ligation-dependent probe amplification by the combination of kits P036, P070, P064, and P250 provided valuable results. Therefore, we suggest that in countries where CMA is still unfeasible as a routine analysis, clinical evaluation followed by karyotyping and MLPA analysis using different kits is still a helpful and affordable solution in particularly within a group of patients with congenital heart defects.

## Materials and Methods

A total of 416 patients were enrolled in this prospective study, including 172 males and 244 females. The age of the patients varied from 8 months to 28 years (mean of 10.5 years).

Inclusion criteria were patients with congenital anomalies, neurodevelopmental delay or intellectual disability, whose G-banding karyotyping from peripheral blood had a normal result or did not explain the clinical findings of the patient. Parental genetic material was also analyzed when necessary.

All patients were clinically evaluated at genetics facilities from five different health centers in Brazil encompassing four different states between 2011 and 2014. All research was performed in accordance with relevant guidelines/regulations. All methods were carried out in accordance with relevant guidelines and regulations. All experimental protocols were approved by the ethics committee of the institution Comissão de Ética para Análise de Projetos de Pesquisa (CAPPesq n° 0282/11), Informed consent forms were obtained from all subjects and signed prior to the participation of all patients in the study by the patients, or by the parents or guardians when participants were under 18 years of age or unable to provide informed consent.

Genomic DNA extraction was performed using 4 mL of peripheral blood with EDTA anticoagulant and the QIAamp DNA Blood Midi Kit (250) (QIAGEN, Valencia, California). DNA samples were investigated by MLPA with the P036, P064, P070, P029, P250 kits.

The kits P036 and P070 were used for the investigation of CNVs in subtelomeric regions, kit P064 was used for detection of more common microdeletions or duplications, kit P029 was used for detection of CNVs within the critical region of Williams-Beuren syndrome (7q11.23), and kit P250 was implemented for detection of CNVs within the critical region of DiGeorge syndrome (22q11.2). MLPA was conducted according to the manufacturer’s recommendations (MRC-Holland®, Amsterdam, the Netherlands).

Results were considered altered when the relative peak was lower than 0.75 (deletion) or higher than 1.25 (duplication) when compared to the control results. Altered results were compared to databases of genomic variants (DGV) (http://projects.tcag.ca/variation/) and DECIPHER (www.decipher.com).

Atypical finds by MLPA in selected cases were investigated using array techniques. In five cases, array assays were performed by CytoScan™ Affymetrix® (Santa Clara, California, USA); in three cases, the CGH-array Agilent SurePrint G3 human genome microarray 180 K (Agilent Technologies, Santa Clara, California, USA) was used; and in six cases, HumanCytoSNP-12 BeadChip (Illumina Inc., San Diego, California) was performed.

Analysis of the variants was performed in accordance with the Database of Genomic Variants and the Database of Genomic Variation and Phenotype in Humans Using Ensembl Resources (DECIPHER).

Patients evaluated by the CGH-array Agilent SurePrint G3 human genome microarray 180 K had results analyzed in collaboration with the Cytogenetic and Cytogenomic Laboratory of the Faculdade de Medicina da Universidade de Coimbra – Portugal (grant FAPESP BEPE n° 2012/25247-6). The others patients were studied in cooperation with the Cytogenomic Laboratory (HC-FMUSP).

All data generated or analyzed during this study are included in this published article.
